# Bone Mineral Density Post a Spinal Cord Injury: A Review of the Current Literature Guidelines

**DOI:** 10.7759/cureus.23434

**Published:** 2022-03-23

**Authors:** Georgia Antoniou, Ioannis S Benetos, John Vlamis, Spyros G Pneumaticos

**Affiliations:** 1 Orthopaedics, Evangelismos General Hospital, Athens, GRC; 2 Orthopaedics, KAT Attica General Hospital, Athens, GRC; 3 Orthopaedic Surgery, KAT Attica General Hospital, Athens, GRC

**Keywords:** prevention of osteoporosis, osteoporotic fractures, skeletal fragility, spinal cord injury, bone mineral density

## Abstract

Background: Spinal cord injury (SCI) causes rapid osteoporosis below the level of injury in a multi-factorial manner. This literature review focused on the early diagnosis of low bone mass (LBM) in SCI patients and aimed to summarize all the available recent data on the diagnosis and treatment of osteoporosis in this unique patient population.

Materials and Methods: Advanced literature research was conducted in the online PubMed database using the keywords 'bone mineral density, 'spinal cord injury, 'skeletal fragility', and 'osteoporotic fractures'. Out of the initial 430 articles, duplicates were removed and the remaining studies were assessed for eligibility. Two reviewers independently extracted data from each study and assessed variable reporting of outcome data. The exclusion criteria were: studies not measuring bone mineral density (BMD), studies comparing SCI to other diseases, animal studies, molecular studies, studies including children, and studies not written in English. The 83 remaining papers were divided into studies focusing on treatment and studies investigating LBM in SCI. Following this step, studies with small patient samples set at 20 patients with SCI for the treatment group and 30 patients for the diagnosis of the LBM group, were also excluded.

Results: In the remaining 32 studies, 18 focused on the diagnosis of LBM in SCI and 14 focused on the various treatment options to address this phenomenon. Most of these studies (n=13) used the dual-energy X-ray absorptiometry (DXA) method to evaluate bone mass while five studies preferred quantitative computed tomography (QCT) measurements and one evaluated LBM using calcaneal qualitative ultrasound. In the treatment group of studies, seven papers administered medication to address LBM and four clinical protocols used physiotherapy methods to reduce bone loss post-SCI while three studies combined medical treatment with physiotherapy.

Conclusion: The unawareness of the unique mechanism through which bone is rapidly lost in the first months post-SCI led to initial scientific confusion. In this review, we summarize information to increase physicians’ awareness of the dangers of ‘silent’ osteoporosis progression post-SCI. We have also provided information on the best timing to evaluate bone loss as well as treatment options that could prevent fragility fractures in this population.

## Introduction and background

Introduction

Spinal cord injury (SCI) causes rapid osteoporosis in a multi-factorial manner through post-injury mechanical disability, neurologic and circulatory dysfunction, and hormonal changes [1,2]. In the first six months post-SCI, bone loss can be as high as 40%. This is especially true for the trabecular bone of lower limbs, making SCI patients susceptible to fractures around the knee joint [3,4]. Unfortunately, these fractures can have devastating consequences such as delayed union or nonunion, autonomic dysreflexia, pressure ulcers, cellulitis, skin breakdown, osteomyelitis or even lower limb amputation which further deteriorate functional impairment and hinder rehabilitation [5-7]. Taking into consideration that bone accumulation is reaching maximum levels at the age of late 20s to early 30s, these injuries, which have been documented to affect primarily young males, come as a shockwave to seize this process and accelerate bone loss [8-10]. With novel technologies and the creation of trauma centers and trauma protocols, specialized teams have managed to salvage many patients with SCI in the recent decade. Nonetheless, patients’ life expectancy and quality of life (QoL) depend on the level of injury and preserved function [11,12]. Part of this QoL is bone health which is sometimes overlooked due to the 'silent' progression of osteoporosis until the emergence of the evident pathologic fracture. As far as clinical practice is concerned, Morse et al. identified diversity in both diagnostic and treatment protocols used to address post-SCI osteoporosis [13]. This literature review focused on the early diagnosis of low bone mass (LBM) in SCI patients and aimed to summarize all the available recent data on the diagnosis and treatment of osteoporosis in this unique patient population.

Materials and Methods

**Figure 1:**
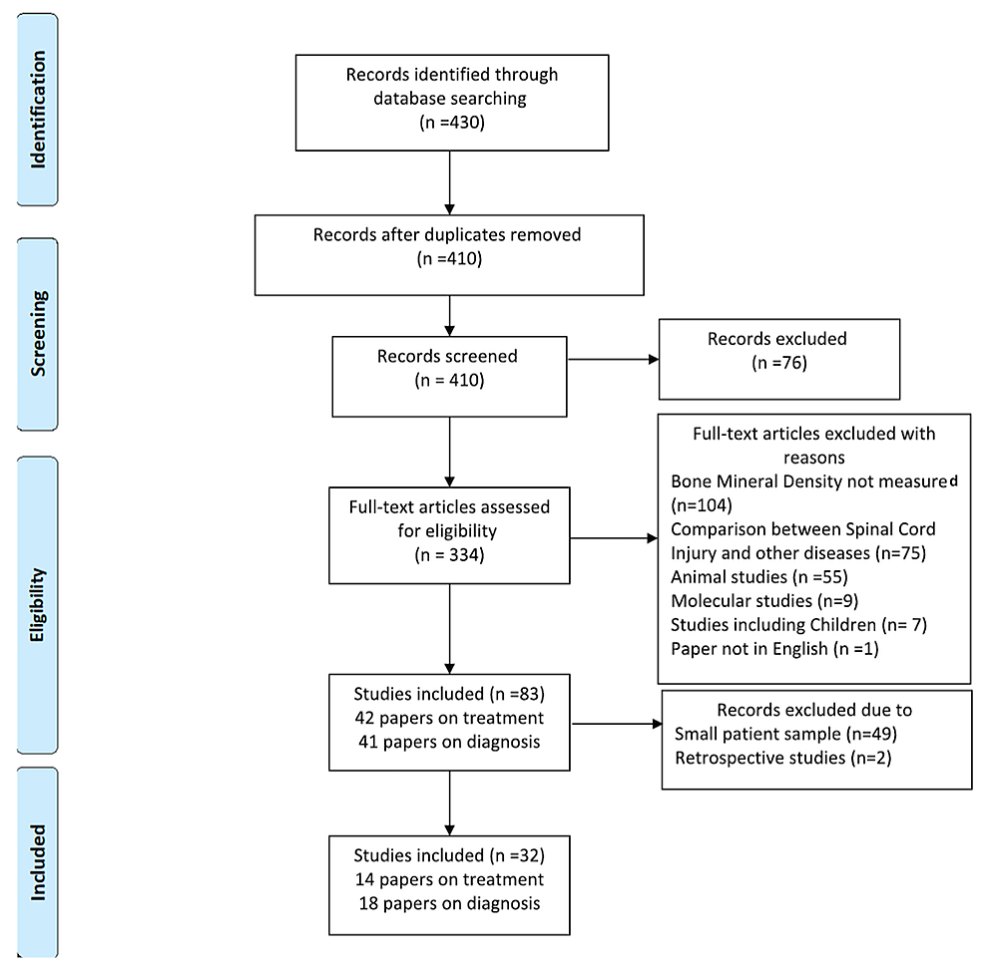
Methodology of literature review

Advanced literature research was conducted by following the Preferred Reporting Items for Systematic Reviews and Meta-Analyses (PRISMA) guidelines and using the online PubMed database to identify records published after 2012 that investigated bone mineral density (BMD) post-SCI. Keywords used were: 'bone mineral density', 'spinal cord injury', 'skeletal fragility' and 'osteoporotic fractures'. Out of the 430 articles identified in the primary research, duplicates were removed and the 410 remaining studies were assessed for eligibility. Two reviewers independently extracted data from each study and assessed variable reporting of outcome data. Exclusion criteria to this review were: studies not measuring BMD (n=104), studies comparing SCI to other diseases (n=75), animal studies (n =55), molecular studies (n=9), studies including children (n= 7), and studies written in languages other than English (n=1). Differences between reviewers were discussed until agreement was achieved. In cases of disagreement, the senior author had the final decision. After the exclusion of 327 studies, the 83 remaining studies were divided into studies focusing on treatment and studies investigating LBM in patients with SCI. Out of the remaining 83 studies, those with small patient samples of 20 patients with SCI for the treatment group and 30 patients for the diagnosis of the LBM group were also excluded. Additionally, two retrospective studies investigating the effect of dietary records and physical activity were also excluded. Finally, 32 studies were included in this review. Eighteen of the 32 studies focused on the diagnosis of LBM in SCI and 14 focused on the various treatment options to address this phenomenon. The process is summarized in Figure 1.

## Review

Results

In the diagnosis group of studies, a total of 1860 patients with SCI were evaluated with the largest series of patients (n=552) being presented by Abderhalden et al. in 2017 [14]. The summaries of these studies are illustrated in Table 1. Most of these studies (n=13) used the dual-energy X-ray absorptiometry (DXA) method to evaluate bone mass while five studies preferred the quantitative computed tomography (QCT) measurements. In one of the studies, Schnitzer et al. examined the effectiveness of calcaneal qualitative ultrasound to evaluate bone mass [15]. As for skeletal areas of SCI patients that were investigated for BMD alterations, 10 studies examined the hip, eight studies examined the knee, eight studies examined the lumbar spine, four studies examined the distal radius, two studies examined the distal tibia, and one study examined the whole skeleton [16]. 

**Table 1:** List of studies investigating bone mineral density in patients with spinal cord injuries in the last decade BMD: Bone mineral density, SCI: Spinal cord injury, dF: Distal femur, pT: Proximal tibia, Scl: Sclerostin, DXA: Dual-energy X-ray absorptiometry, QCT: Quantitative computed tomography (QCT), U/S: Ultrasound, TH: Total hip, LS: Lumbar spine, FN: Femoral neck, Fx: Fractures, TB: Total body, FE: Finite element, 25OH Vit D: 25hydroxy vitamin D, pF: Proximal femur, dT: Distal tibia

Author	Number of patients with SCI	Intervention	Results
Schnitzer et al. 2012 [15]	66, 20 acute and 46 chronic	Qualitative U/S calcaneus/ DXA TH, LS, FN	•Qualitative U/S of the calcaneus effective, quick, no irradiation for patients •Rapid bone loss post SCI regardless of patient's age, gender and severity of motor involvement
Morse et al. 2012 [17]	39 chronic	DXA scans dF, pT and radius in SCI patients and controls Scl levels	•Lower BMD dF, pT in wheelchair-dependent patients •Scl levels positively associated with BMD dF, pT but not radius
Doherty et al. 2014 [18]	149 chronic	DXA pT, dF, hip and radius	•Wheelchair-dependent patients with lower BMD around the knee and hip, more likely to have osteoporosis •Increased Fx risk at all sites in wheelchair-dependent patients as compared to walking patients with SCI
Javidan et al. 2014 [19]	148 chronic	DXA LS, pF, TH	•Osteopenia in both genders at pF not LS •Males have lower BMD at all sites compared to women
Sabour et al. 2015 [20]	140 acute	DXA TH, pF, LS Leptin levels	•More prominent reduction of BMD in pF compared to LS •Lesions above T6 cause higher BMD reduction especially if autonomic dysreflexia co-exists •Leptin associated with female BMD at TH and pF
Kostovski et al. 2015 [16]	31 acute	DXA scans LS, pF and TB Bone turnover markers	•Cortical bone loss primarily through endosteal resorption •Exponential decrease in torsional stiffness and strength at pT the first two years post SCI
Edwards et al. 2015 [3]	60 chronic	QCT pT and FE analysis	•Greater bone loss in the epiphyseal region
Gibbs et al. 2015 [21]	70 chronic	QCT of distal lower extremities for bone and muscle density	•Muscle size correlates with tibial bone size and geometry in SCI •Muscle density associated with trabecular bone BMD
Abderhalden et al. 2017 [14]	552 chronic	DXA hip and LS	•Hip T scores lower than LS •LS T score did not predict Fx risk •Nearly 50% had osteoporosis
Haghighat Khah et al. 2018 [22]	44 chronic	DXA and QCT LS	•No significant superiority of QCT compared to DXA for LS assessment
Haider et al. 2018 [2]	101	QCT around the knee and FE analysis	•Steady state of bone loss at 3 to 5 years post SCI •Axial and torsional stiffness 40% to 85% lower in acute SCI compared to chronic SCI •No age-related bone loss
Cirnigliaro et al. 2019 [23]	105	DXA around the knee and hip	•Loss of BMD continuous in the second decade after SCI even in regions with predominant trabecular bone dF or pT
Maïmoun et al. 2019 [24]	131, 23 acute and 108 chronic	DXA LS, pF and radius in SCI Periostin, sclerostin and bone turnover markers	•Lower BMD pF in SCI patients •LS BMD not altered in SCI patients •SCI patients have significantly higher periostin and lower sclerostin levels in the acute phase
Frotzler et al. 2020 [25]	43	DXA hip QCT pT and tibial diaphysis	•Lower BMD at all areas in SCI patients compared to controls except distal tibia epiphysis •Higher Fx rate in SCI patients
El-Kotob et al. 2021 [26]	70 chronic	QCT dT and tibial diaphysis	•Higher cortical bone than trabecular bone BMD loss •No association between cortical bone BMD and muscle density
Ghasem-Zadeh et al. 2021 [27]	31	QCT dT, fibula and radius	•Lower BMD at dT and fibula in SCI patients •Radius BMD not altered •Trabecular thickness increased in SCI compared to controls
Choi et al. 2021 [28]	44	DXA pF and LS	•No significant correlation between LS BMD and duration from injury •Significant decrease in pF BMD, especially femoral neck with increased duration of time post SCI
Zheng et al. 2021 [29]	36 acute	DXA dF, pT and hip	•All patients had lower BMD at all sites compared to controls •At six weeks significantly lower BMD at pT compared to controls •Hip BMD decreased later (at three months post SCI) compared to dF and pT •Age and 25OH Vit D influenced dF BMD •Age and gender influenced pT BMD

In the treatment group of studies, a total of 608 patients were treated for LBM post SCI. In seven studies, patients received anti-resorptive medication such as zoledronic acid (ZA), denosumab and teriparatide. In a single study by Hatefi et al. in 2018, the clinicians addressed LBM with curcumin [30]. As for the rest of the studies, four clinical protocols used physiotherapy methods to reduce bone loss and three studies combined medical treatment with physiotherapy. The summaries of these studies are illustrated in Table 2.

**Table 2:** List of studies treating low bone mass in individuals with spinal cord injury within the last decade BMD: Bone mineral density, pT: Proximal tibia, dF: Distal femur, LS: Lumbar spine, pF: Proximal femur, FES: Functional electrical stimulus, FN: Femoral neck, TH: Total hip, RANKL: NF-kappaB ligand

Author	Number of patients with SCI	Treatment	Duration of treatment in months	Results
Meng et al. 2014 [31]	40 acute	Oral calcium + standing in electrical bed for 30 minutes twice a day + massage + pulse magnetic field treatment +/- traditional Chinese acupuncture and moxibustion	3	•No statistically significant result of acupuncture to BMD
Dudley Javoroski et al. 2016 [32]	42 chronic	Vibration treatment while seated in wheelchair 3 times per week	12	•No statistically significant result of vibration to BMD •No retention of trabecular bone architecture in pT and dF
Gifre et al. 2016 [33]	23 acute	Denosumab 6o mg	6	•Denosumab preventing sublesional bone loss •Undetectable RANKL levels at pF after denosumab
Craven et al. 2017 [34]	34 chronic	FES therapy or conventional aerobic and resistance training for 45 minutes 3 times per week	4	•Singificant increase in osteocalcin with FES but no effect in actual bone strength
Hatefi et al. 2018 [30]	100	Curcumin 110 mg adjusted to the weight of the patient per day	6	•Significant decrease in osteoporosis progression at the LS, FN and hip with curcumin treatment
Edwards et al. 2018 [35]	61 chronic	Teriparatide 20 μg per day + sham vibration for 10 min per day or vibration alone or teriparatide 20 μg per day + vibration	12	•At 12 months of treatment teriparatide managed to increase LS BMD but not hip BMD regardless of vibration treatment
Goenka et al. 2018 [36]	60 acute	Zoledronic acid 5 mg	12	•Significant decrease of BMD at FN and TH
Rodriguez et al. 2019 [37]	30 chronic	Cardiorespiratory fitness		•No direct correlation between cardiorespiratory fitness and bone health •High cardiorespiratory fitness maintains arm bone health
Oleson et al. 2020 [38]	32 acute	Zoledronic acid 5 mg	12	•At 4 months post-treatment dF and hip BMD were increased but this effect was lost at 12 months of treatment •pT BMD was not affected
Goenka et al. 2020 [39]	60 acute	Zoledronic acid 5 mg	12	•Zoledronic acid at 12 months effective in preventing forearm bone loss
Cirnigliaro et al. 2020 [40]	26 subacute	Denosumab 60 mg	12	•Maintenance of BMD around the knee with denosumab treatment
Fang et al. 2021 [41]	20	FES rowing exercise +/- a single dose of zoledronic acid	12	•Combination of FES and zoledronic acid had longer effect on dF than pT •Zoledronic acid not so effective on trabecular bone
Holman et al. 2021 [42]	20 chronic	Testosterone +/- resistance training for 16 weeks	4	•Combination of testosterone and resistance treatment decreases yellow bone marrow adiposity and increases trabecular bone parameters
Edwards et al. 2021 [43]	60 acute	Zoledronic acid 5 mg for the first year +/- second dose the second year	24	•A single dose of zoledronic acid preserves pF, dF and pT BMD and is well tolerated with no side effects

Discussion

Mechanism of Bone Loss

Bone loss after SCI has been well-documented in the literature during the last decades. In an attempt to recognize the exact mechanism under which this phenomenon is evolving, many clinical studies were assessed during this literature research. However, only studies including a significant number of patients were selected. The presence of a unique pattern of bone loss that cannot be correlated with other forms of bone loss has been highlighted by Battaglino et al. in 2012. The authors concluded that SCI is related to a multifactorial and unique progression of sublesional osteoporosis [44]. Bone resorption due to mechanical disuse may be easily understood. However, the combination of this mechanical parameter to hypothalamic disturbances, adipose tissue accrual, insulin resistance and spasticity needs to be taken into serious consideration [45,46]. 

In the acute setting post-SCI, the osteoblastic activity is suppressed and excessive bone resorption is accompanied by hypercalcemia, hypercalciuria and increased serum bone resorption markers such as alkaline phosphate and osteocalcin [47]. This process reaches a maximum rate at 10 to 16 weeks post-injury and affects both the trabecular and cortical bone [48]. Muscle loss accompanies bone loss with muscle volume being associated with tibial bone size and geometry post an SCI. However, the pathophysiologic mechanism of this process remains unidentified [21,49]. Charmetant et al., in a literature review in 2010, noted that this process reaches a steady state at one year post an SCI [50]. However, according to a more recent study by Edwards et al. in 2015, this process seems to be long lasting reaching a steady state at two to one, or two to seven years post-injury [3]. In another recent study by Haider et al, including 101 patients, this steady-state was set at 3.5 years [2]. The most devastating consequences of this process are found around the knee where BMD reduction reach rates of up to 50% [18,50]. In a large series by Abderhalden et al., including 552 veterans with SCI, at two years post-SCI half the patients were diagnosed with osteoporosis [14]. Thus, our therapeutic window to effectively prevent bone loss may be within the first two years post-SCI.

Factors Affecting Bone Loss

An interesting finding of this review is that the rate of bone loss post-SCI is neither associated with a patient's age nor gender but rather associated with time from injury [17,19]. Furthermore, bone loss is affected by injury levels with wheelchair-dependent patients demonstrating lower levels of BMD compared to walking patients with SCI [28, 29]. In a single study by Javidan et al. including 149 patients, males seemed to have lower BMD at all skeletal sites compared to females [19]. However, the distribution of gender in this study was uneven (32 females to 116 males). Studies with a better equivalence between the genders are required to further investigate the possibility of men being more sensitive to bone loss post-SCI. 

Primarily Affected Skeletal Regions

As for the skeletal regions most frequently affected, significantly lower BMD at the proximal tibia was measured in 36 patients as soon as six weeks after SCI by Zheng et al. in 2021 [29]. The same authors reported that hip BMD is affected later by approximately three months post-injury, while Choi et al., in the same year, reported that the femoral neck region is most prone to post-SCI osteoporosis [28]. A greater loss is measured in all studies at the epiphyseal region of bone with the cortical bone loss being higher than trabecular bone loss [26]. This bone loss is a result of endosteal bone resorption, as highlighted by Kostovki et al. [16]. Another interesting finding of this review is the unaffected BMD of the lumbar spine regardless of time post-SCI [16,19,20]. Furthermore, lumbar spine BMD measurement failed to predict fracture risk in a large study by Abderhalden et al. [14]. This phenomenon could be partially explained by the distribution of weight through the spine in a prolonged sitting position and wheelchair usage [24,51]. All the above data come into accordance with the updated International Society of Clinical Densitometry (ISCD) guidelines of 2019 stating that DEXA measurements in SCI patients should include the hip, distal femur and proximal tibia regions but not the spine [52]. Quantitative computed tomography may work as an alternative option given the unique advantage of finite element analysis and separate examination of trabecular and cortical BMD [53]. However, this technique has its own technical demands and failure of the exact positioning of the slice cuts at 3 mm may lead to wrong measurements of BMD [54].

Treatment Methods

For the treatment of LBM in SCI two major categories exist, pharmacological therapy and physical therapy. In this literature review, patient samples were small in many studies and a cut off value of 20 patients was applied to consider patient sampling adequate. The anti-osteoporotic medical regimens used were zoledronic acid, denosumab, teriparatide and curcumin in both the acute and chronic phase of SCI. Zoledronic acid (ZA) is a bisphosphonate and is the most commonly administered medication in SCI patients alone or in combination with other treatments. Five milligrams of ZA seemed to be effective in reducing BMD loss in the femoral neck and total femur area as Goenka et al. noted in their study that included 60 patients with acute SCI [36]. The same authors took their study a step further in 2021 demonstrating the positive effect of ZA in preventing bone loss in the forearm region [39]. Even in the protocol of Fang et al.'s study where a combination of ZA and functional electrical stimulus (FES) rehabilitation protocol was used, FES alone failed to prevent bone loss, highlighting the importance of pharmacological treatment [41]. Regardless of these promising results, the therapeutic action of ZA seems to reach a plateau at 12 months with the clinical research of Oleson et al. in 2020 demonstrating that at one year of treatment it is no longer as effective as in the first four months [38]. Regardless of these new data, due to the broad usage of bisphosphonates in all types of osteoporosis, ZA remains the first option for clinicians for the treatment of osteoporosis in SCI. A promising pharmacological agent in the treatment of LBM post-SCI is denosumab, a monoclonal antibody with high linkage to NF-kappaB ligand (RANKL). Two studies, including 23 and 26 patients in the acute and subacute phase post SCI, respectively, were found in the current literature. Denosumab managed to prevent sublesional bone loss and maintained BMD around the knee in the clinical studies by Gifre et al. in 2017 and Cirnigliaro et al. in 2020 [33,40]. However, their patient samples were not as large as those included in ZA clinical protocols and the effect of denosumab was only studied in the first year after SCI. As a result, larger series with longer follow-ups are required to assess the effectiveness of denosumab in SCI. Additionally, comparative studies between ZA and denosumab with a sufficient number of patients are lacking in current literature and could offer important information for choosing the best pharmacologic agent in both the acute and chronic phase of SCI.

As for the rehabilitation protocols suggested, unfortunately, most studies in the current literature included small patient samples. However, in this review, only the largest series were included (see above in Table 2). Acupuncture and vibration therapy alone did not seem to have a significant impact on BMD [31,32]. Even when vibration was combined with teriparatide it was underlined that the pharmacological agent was effective regardless of the vibration treatment [35]. As for cardiorespiratory fitness (CRF) rehabilitation protocols, no direct relationship was found between them and bone health. However, high-intensity CRF seemed to maintain BMD in the arms according to clinical research by Rodriguez-Gomez et al. in 2019 [37]. Functional electrical stimulus showed the most promising results in two series by Dudley-Javoroski et al. in 2012 [55] and Chen et al. in 2005 [56]. But in newer clinical studies with larger patient samples such as the one by Craven et al. in 2017, these results were questioned with the authors concluding that FES may affect laboratory values of bone turnover markers but has no actual effect on bone strength [34]. When FES is combined with ZA, prevention of bone loss at the distal femur seems to last longer than the prevention provided by ZA treatment alone [41]. As a result, even though rehabilitation protocols do not seem to be particularly effective in preventing bone loss, their combination with pharmacological agents could offer long-lasting protection, which could benefit patients with SCI.

**Figure 2:**
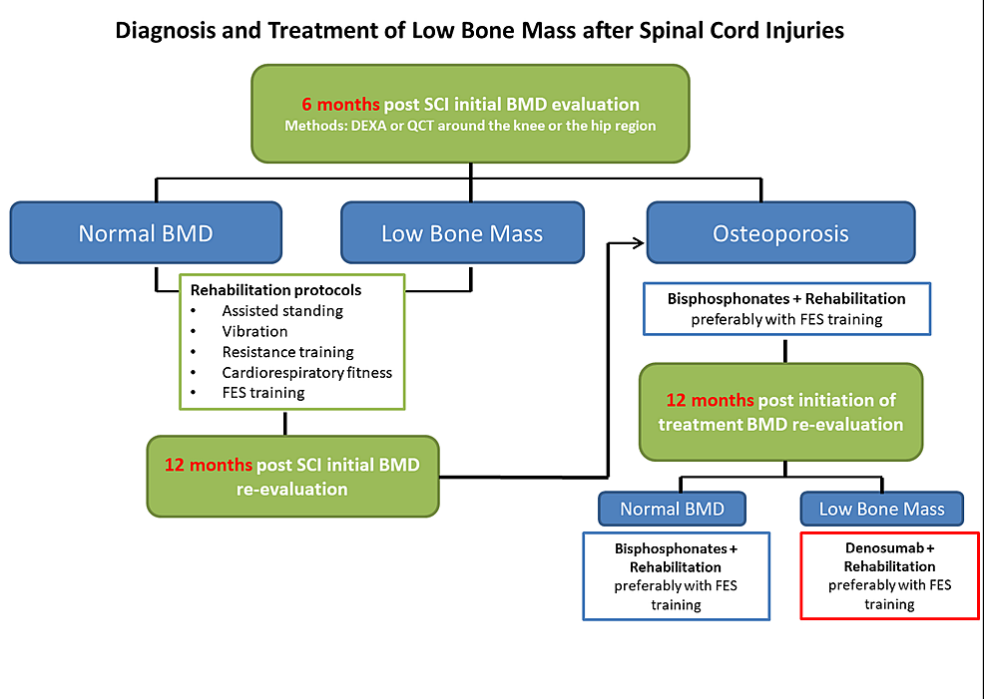
Suggested diagnosis and treatment approach algorithm for low bone mass after spinal cord injuries SCI: Spinal cord injury, BMD: Bone mineral density, FES: Functional electrical stimulus

Taking into consideration that a DEXA is required before initiating treatment and by knowing, based on the results of this review, that within the first two years there is a good therapeutic window to treat LBM in SCI, we would suggest the first semester as the ideal timing for the first evaluation. For patients with established LBM, physiotherapy protocols should be initiated not only for the bone to be preserved but also for the cardiovascular benefits they offer [57,58]. In case there is osteoporosis, antiresorptive treatment with bisphosphonates should be initiated with a combination of rehabilitation protocols such as FES which has been shown to have the best results when compared to other protocols. After a year of treatment, a second evaluation should take place to verify that bisphosphonates are preventing bone loss. Based on the results of this review, in case of bone loss even while on bisphosphonate treatment, the initiation of denosumab should be considered. If there is normal bone mass in the first-semester post-injury, patients should be re-evaluated with a new DEXA scan a year after the injury. In this second examination, if the rapid progression of bone loss is present, antiresorptive treatment should be initiated even if the osteoporosis criteria are not yet met. This treatment algorithm is summarized in Figure 2 and is our suggestion based on the results of this review.

## Conclusions

The purpose of early diagnosis of LBM in patients who have suffered an SCI is the prevention of pathologic fractures and their devastating complications. The lack of awareness of the unique mechanism through which bone is rapidly lost in the first months post-SCI led to initial scientific confusion. The first clinical studies attempted to use lumbar spine DEXA protocols utilized in post-menopausal women to assess patients with SCI. However, it was soon realized that this bone loss was primarily sublesional and did not affect the lumbar spine. According to the revised ISCD guidelines of 2019, lumbar spine BMD does not predict fracture risk in SCI while the hip and knee regions are better representatives of bone health in this population. What is yet to be answered is the ideal timing of the first DEXA evaluation after SCI. Based on the results of this review we have provided a treatment algorithm as a first step to aid physicians in this field. 

Future research is expected, with our review only attempting to provide sufficient information and updated guidelines. Unfortunately, the large heterogeneity of the studies encountered in the current literature did not allow for meta-analyses to be conducted. Regardless of this step back, we believe that we summarized enough information to increase awareness in physicians of the dangers of ‘silent’ osteoporosis progression in SCI. We have also provided information on the best timing to evaluate bone loss as well as treatment options that could prevent fragility fractures in this population. 
